# Enhanced Detection of Recurrent Diffuse Malignant Peritoneal Mesothelioma Using ^68^ Ga-FAPI PET/CT Compared to ^18^ F-FDG PET/CT: A Case Report

**DOI:** 10.1055/s-0045-1805043

**Published:** 2025-03-12

**Authors:** Marwah Abdulrahman, Ula Al-Rasheed, Ali Dabous, Akram Al-Ibraheem

**Affiliations:** 1 Department of Nuclear Medicine, King Hussein Cancer Center (KHCC), Amman, Jordan; 2 Department of Radiology and Nuclear Medicine, School of Medicine, the University of Jordan, Amman, Jordan; 3 Department of Transplant and Oncosurgery, King Hussein Cancer Center (KHCC), Amman, Jordan

**Keywords:** FAPI, DMPM, malignant peritoneal mesothelioma, ^68^ Ga-FAPI PET/CT, FAPI theranostics

## Abstract

Diffuse malignant peritoneal mesothelioma (DMPM) is a rare and aggressive subtype of epithelioid mesothelioma that arises from the lining of the abdominal cavity. While the applications of traditional fluorine-18 fluorodeoxyglucose ( ^18^ F-FDG) positron emission tomography/computed tomography (PET/CT) in diagnosis and staging of DMPM are well-established, the utility of gallium-68 fibroblast activating protein inhibitor ( ^68^ Ga-FAPI) PET/CT in detecting disease recurrence remains an area that requires further research and validation, with limited literature. Implementing FAPI PET/CT for these cases may provide superior lesion detectability and higher reporter confidence, prompting the need for further studies to investigate the potential future role of FAPI theranostics in guiding treatment decisions for DMPM. This case report describes a 49-year-old male patient diagnosed with DMPM, who underwent cytoreductive surgery and hyperthermic intraperitoneal chemotherapy but developed recurrent disease that was better visualized on ^68^ Ga-FAPI PET/CT compared with ^18^ F-FDG PET/CT.

## Introduction

Primary diffuse malignant peritoneal mesothelioma (DMPM) is an uncommon and highly invasive type of malignant mesothelioma, comprising approximately 20 to 30% of all mesothelioma cases, with an overall incidence of approximately one to two cases per million individuals. 1 DMPM is characterized by the widespread presence of multiple small nodules or plaques originating from the peritoneal surface, mesentery, or omentum. 2 Notably, only approximately one-third of patients diagnosed with DMPM had a history of asbestos exposure, making it a distinct entity from other types of mesothelioma. 3

For primary diagnosis, the gold standard imaging technique is the computed tomography (CT) scan, which effectively detects mesenteric and peritoneal thickening. 4 However, the integration of fluorine-18 fluorodeoxyglucose ( ^18^ F-FDG) positron emission tomography/CT (PET/CT) has revolutionized DMPM diagnosis through quantitative standardized uptake value (SUV) analysis to distinguish benign from malignant peritoneal lesions, improving the diagnostic approach and treatment planning with superior specificity, sensitivity, and accuracy rates of 92, 87, and 86%, respectively. 5

Nevertheless, this modality sometimes falls short in identifying non-FDG-avid or low attenuation peritoneal deposits. 4 Thus, fibroblast activating protein inhibitor (FAPI) PET/CT may yield augmented diagnostic outcomes with higher SUV _max_ and tumor-to-background ratios (TBRs) in both primary lesions and lymph node metastases. 6

In this study, we are the first to report a case of DMPM where recurrent peritoneal deposits were nonhypermetabolic on FDG-PET but showed avidity on FAPI imaging, aiding in the verification of disease recurrence.

## Case Presentation

**Fig. 1:**
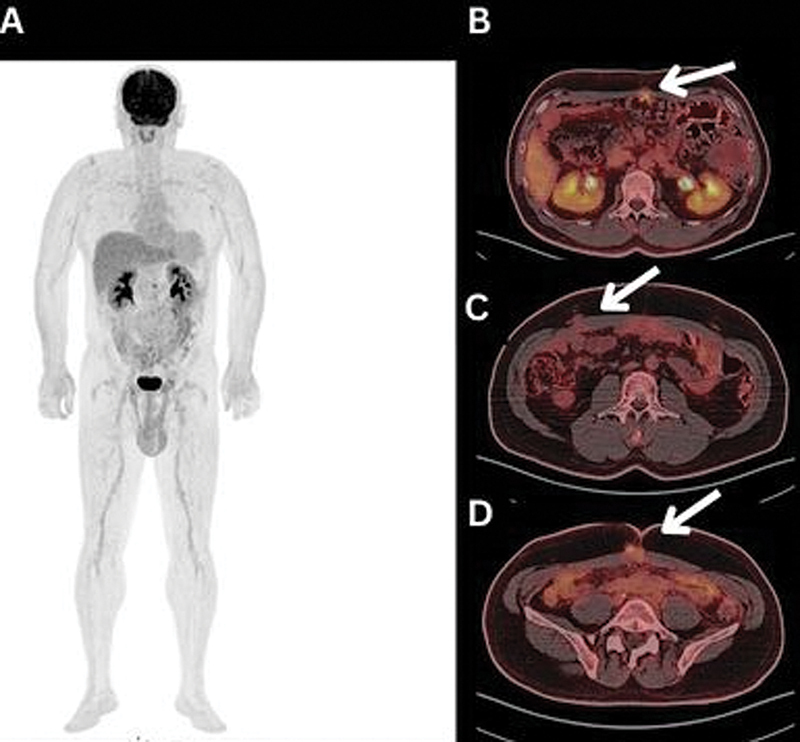
Follow-up fluorine-18 fluorodeoxyglucose ( ^18^ F-FDG) positron emission tomography/computed tomography (PET/CT) whole body scan was performed post-laparotomy, omentectomy, complete peritoneal stripping, bilateral diaphragm stripping, repair cholecystectomy, and hyperthermic intraperitoneal chemotherapy (HIPEC). ( **A** ) Maximum intensity projection (MIP) and ( **B** – **D** ) axial fused PET/CT images detected a few soft tissue thickenings/nodules at the anterior median/paramedian areas of the anterior abdominal wall, showing mild increased FDG uptake (white arrows), the most prominent one ( **B** ) measured ∼0.75 cm in the longest axial diameter (LAD) with maximum standardized uptake value (SUV _max_ ): 4.97, reported as omental deposits.

**Fig. 2:**
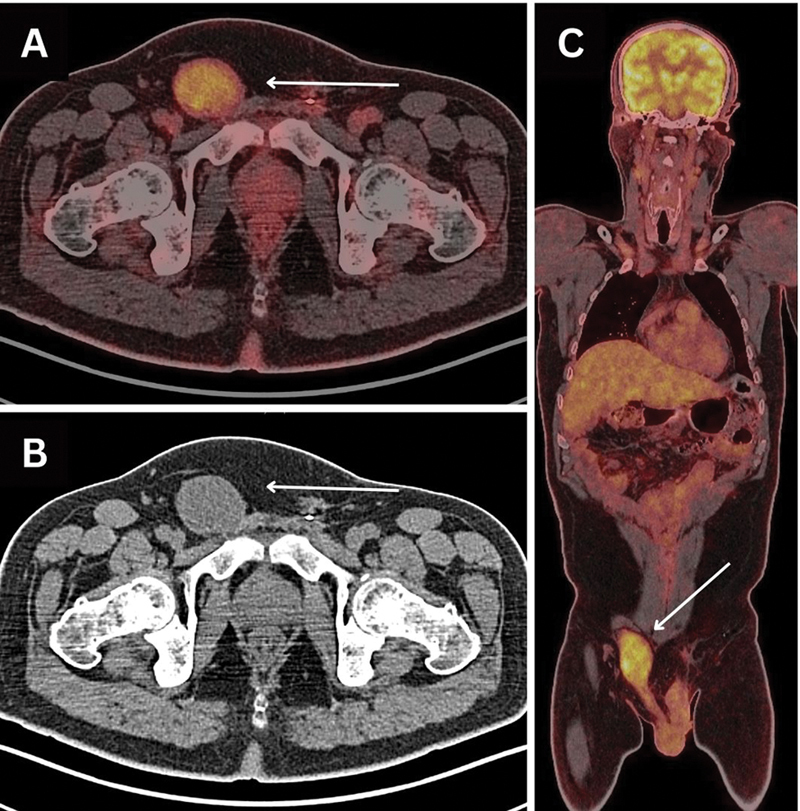
Whole-body positron emission tomography/computed tomography (PET/CT) scan by fluorodeoxyglucose (FDG) ( **A** and **C** , fused images in axial and coronal planes, respectively) revealing interval development of hypermetabolic right inguinal soft tissue mass, running with the course of upper spermatic cord reaching the external iliac region (white arrows), showing increased FDG uptake, measuring ∼4.7 × 3.5 × 7.0 cm with an maximum standardized uptake value (SUV _max_ ): 5.1. ( **B** ) CT component of the scan.

**Fig. 3:**
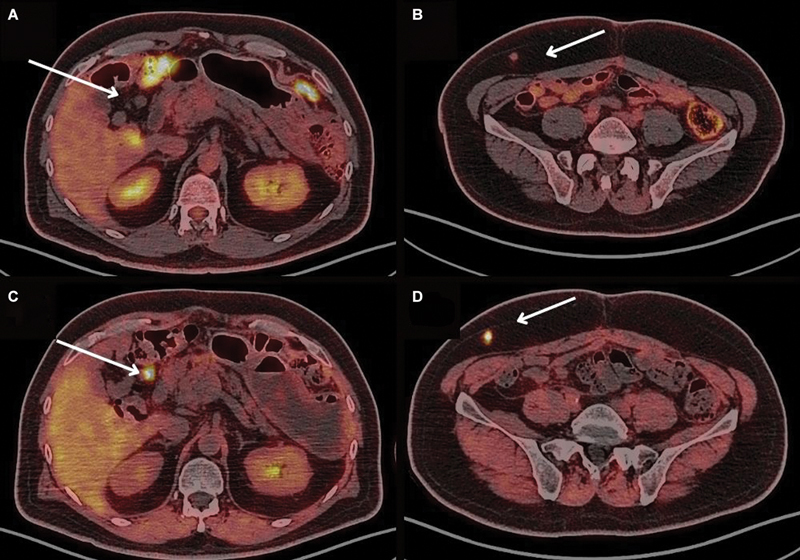
Gallium-68 fibroblast activating protein inhibitor ( ^68^ Ga-FAPl) positron emission tomography/computed tomography (PET/CT) was requested to confirm disease recurrence, comparison was made with fluorine-18 fluorodeoxyglucose ( ^18^ F-FDG) PET/CT (1a and 1b) axial fused images showing non-FDG-avid areas of subcutaneous, peritoneal, and omental nodules (white arrows), high pathological FAPI uptake (2a and 2b) was noted in the same lesions with an maximum standardized uptake value (SUV _max_ ) up to 14.5 compared with a prior SUV _max_ of only 1 in FDG PET, with a stable measurement of 0.7 cm in maximal axial diameter (white arrows).

**Fig. 4:**
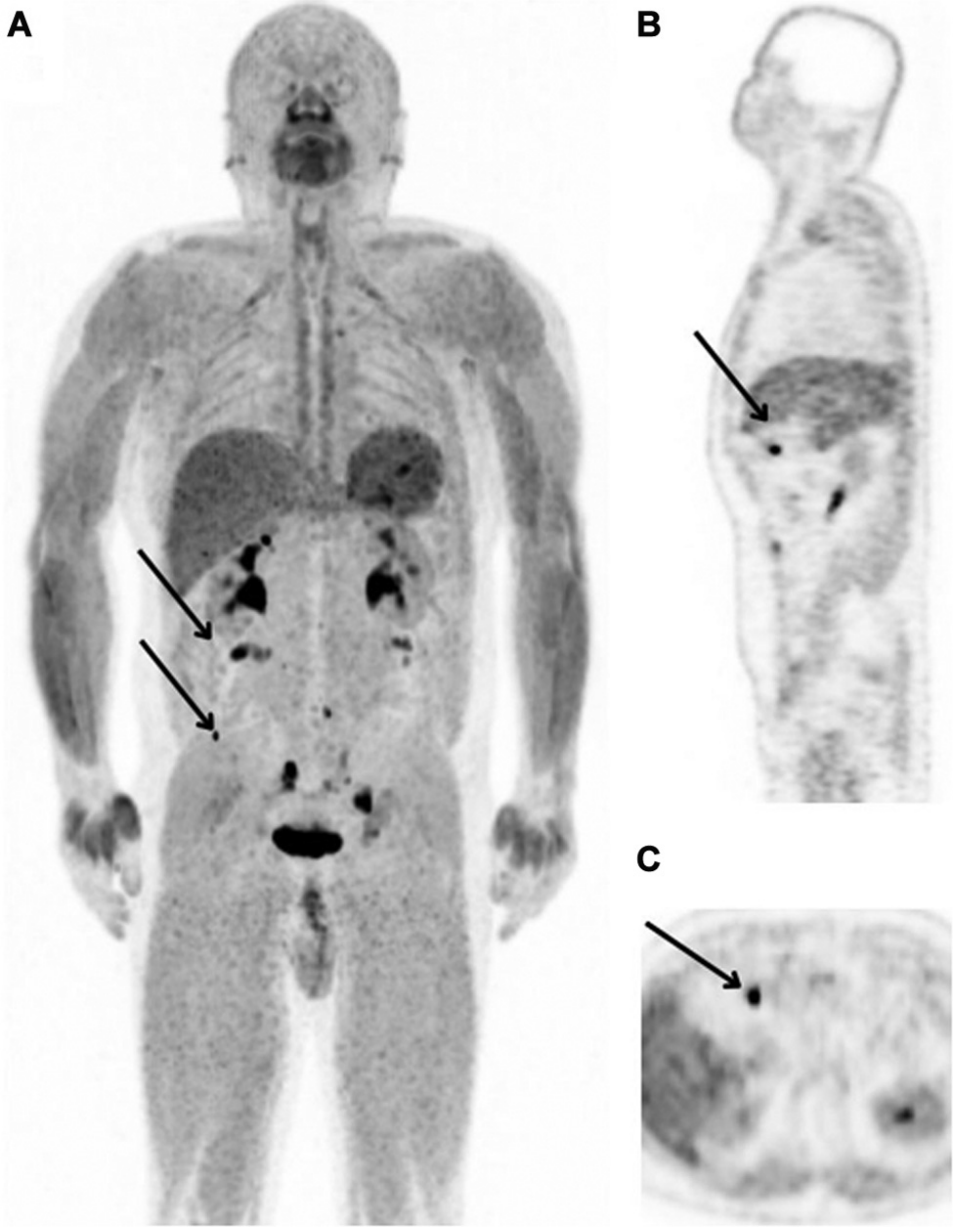
Maximum intensity projection (MIP) ( **A** ), positron emission tomography (PET) sagittal ( **B** ), and axial ( **C** ) planes of the gallium-68 fibroblast activating protein inhibitor ( ^68^ Ga-FAPI) PET/computed tomography (CT) scan showing elevated uptake in suspicious lesions (black arrows). MIP, maximum intensity projection.

A 49-year-old single male patient was admitted to our hospital complaining primarily from a gradual onset of abdominal distension and discomfort. The patient had type 2 diabetes mellitus and hypertension that were well-controlled on medications, with an unremarkable past surgical history and a negative familial cancer history. He stated working in an office environment with no exposure to any isolation materials. Despite experiencing occasional nausea over the past 3 months, he did not report any instances of fever, vomiting, or weight loss. Physical examination findings included mild abdominal tenderness, shifting dullness on percussion, and reduced bowel sounds on auscultation. The initial workup included biochemical tests encompassing a complete blood count, renal function test, liver function test, and serum electrolytes, all were within normal ranges, in addition to the following tumor marker tests: cancer antigen 19–9 (CA 19–9): 7.6, CA 125: 7.6, and carcinoembryonic antigen: 0.7. Diagnostic imaging included chest X-ray that showed no lung abnormalities, such as pleural thickening or pleural effusion. An abdominopelvic CT scan revealed diffuse omental and peritoneal nodularity and thickening, particularly in the right upper abdomen, along with moderate free fluid and a mildly enlarged celiac lymph node (up to 1 cm). Laparoscopic diagnostic peritoneal biopsy disclosed atypical mesothelial proliferation in favor of diffuse peritoneal epithelioid mesothelioma. Accordingly, the patient underwent cytoreductive surgery (omentectomy + peritonectomy + cholecystectomy + diaphragmatic stripping + small bowel and large bowel mesenteric stripping + the epiploic appendices) and hyperthermic intraperitoneal chemotherapy with mitomycin. Histopathological examination of the resected tissue verified the presence of multifocal epithelioid mesothelioma. Postsurgical follow-up with ^18^ F-FDG PET/CT was ordered within 3 months demonstrating multiple hypermetabolic nodular soft tissue thickenings located in the anterior median and paramedian regions of the abdominal wall, suggestive of peritoneal and omental deposits, potentially indicating local disease recurrence ( Fig. 1 ). A follow-up ^18^ F-FDG PET/CT scan revealed a hypermetabolic right inguinal soft tissue mass along the upper spermatic cord extending to the external iliac region. This led to a curative right inguinal orchidectomy with spermatic cord excision, which confirmed testicular mesothelioma ( Fig. 2 ). However, the patient was in remission until the latest follow-up assessment with FDG PET/CT exhibiting a new small right lower abdominal subcutaneous nodule with minimal to non-FDG uptake, with additional few suspicious peritoneal-based deposits that did not show significant metabolic activity. Therefore, gallium-68 ( ^68^ Ga)-FAPI PET/CT was requested for a more comprehensive assessment. Interestingly, compared with ^18^ F-FDG, ^68^ Ga-FAPI had conclusively verified the presence of disease recurrence showing increased FAPI avidity with a SUV _max_ up to 14.5 ( Figs. 3 and 4 ). Multidisciplinary committee decided on six cycles of carboplatin and pemetrexed, followed by ^68^ Ga-FAPI PET/CT-guided follow-up.

## Discussion

Fibroblast activation protein (FAP) is a type II transmembrane serine protease, recognized to be overexpressed in the stromal tissue of various epithelial cancers. This protein is primarily found in cancer-associated fibroblasts (CAFs), which are a hallmark of the tumor microenvironment. 7 Mesenchymal tumors, such as sarcomas and mesotheliomas, are recognized to exhibit intense uptake of FAPI due to their histogenetic origins. 8 This is attributed to the significant presence of CAFs in these tumors. 9 DMPM is a rare primary malignancy that originates from mesothelial cells in the peritoneum, with its unique characteristic of a complex interaction between mesothelial cells and mesenchymal cells, particularly fibroblasts and myofibroblasts, involving a mesothelial-to-mesenchymal transition, where mesothelial cells convert into myofibroblast-like cells. 10 This transition contributes to the tumor microenvironment and the generation of CAFs with high FAP expression, which are critical for tumor progression and metastasis, leading to the aggressive behavior and poor prognosis of DMPM. 10 PET imaging sensitivity for detecting small lesions is expected to be higher when targeting tumor stroma than glycolysis, as the stromal compartment of a tumor can often exceed the volume of the tumor itself, providing enhanced metastatic depiction. 11 Hence, FAPI-labeled tracers like ^68^ Ga have the potential to address the possible limitations associated with ^18^ F-FDG and may be more effective in detecting peritoneal infiltration with DMPM, which occasionally exhibit low FDG avidity. ^68^ Ga-FAPI is characterized by minimal physiological uptake in normal tissue such as the bowel, 12 enhancing TBR contrast for clearer visualization of small peritoneal lesions. 13 A case report described the diagnostic challenges in a patient with an undetected primary tumor who underwent ^18^ F-FDG PET/CT, revealing non-FDG-avid peritoneal thickening. Subsequent ^68^ Ga–FAPI PET/CT showed high uptake, confirming malignant peritoneal mesothelioma via tru-cut biopsy. 12 Similarly, in a case study of malignant peritoneal mesothelioma exhibiting widespread butterfly-shaped muscle metastasis, researchers observed that ^68^ Ga-FAPI PET/CT provided a clearer visualization of relevant lesions compared with ^18^ F-FDG PET/CT. 13

The use of therapeutic radionuclides like lutetium-177 ( ^177^ Lu) labeled to FAPI molecules allows for precise targeting of tumors that overexpress FAP, enhancing internal radiation therapy. Ongoing research efforts are expected to uncover the full potential of FAPI as an innovative imaging tool and pave the way for its integration into simultaneous targeted radioligand therapy. 14 15

## Conclusion

Although the definitive diagnosis of DMPM is typically made through histological examination of tissue samples, the advent of molecular imaging techniques has opened new avenues for the detection, staging, and monitoring of these tumors. One such promising development is the use of FAPI in PET imaging.
